# Occupational and lifestyle risk factors associated with metabolic fatty liver disease among university staff in Indonesia

**DOI:** 10.3389/fpubh.2025.1739931

**Published:** 2025-12-16

**Authors:** Mutmainnah Husaema, Lalu Muhammad Saleh, Muhammad Furqaan Naiem, Atjo Wahyu, Darmawansyah D

**Affiliations:** 1 Master of Occupational Health and Safety Program, Hasanuddin University, Makassar, Indonesia; 2 Department of Occupational, Hasanuddin University, Makassar, Indonesia; 3 School of Postgraduate Studies, Hasanuddin University, Makassar, Indonesia

**Keywords:** dyslipidemia, employees, MASLD, obesity, sex differences, years of service

## Abstract

Fatty liver disease, now classified under the metabolic dysfunction-associated steatotic liver disease (MASLD) spectrum, has become increasingly common among working populations due to the rising prevalence of obesity, dyslipidemia, and metabolic disorders. This study examined the associations between fatty liver severity and individual as well as occupational factors among university employees. A cross sectional study was conducted among 78 staff members using data from the 2024 Medical Check-Up records. Variables collected from medical records and structured questionnaires included age, sex, obesity, dyslipidemia, diabetes mellitus, hypertension, smoking, alcohol and coffee consumption, exercise habits, employment status, years of service, and work unit. Data were analyzed using Chi-square and multivariate logistic regression tests. The majority of participants had mild fatty liver (73.1%), while 26.9% had moderate to severe grades. Bivariate analysis revealed significant associations with sex, obesity, dyslipidemia, and years of service. In multivariate analysis, male sex (OR = 10.17; *p* = 0.002), dyslipidemia (OR = 18.30; *p* = 0.010), and obesity (OR = 6.36; *p* = 0.049) were identified as dominant predictors. These findings highlight that metabolic risk factors and occupational duration contribute to fatty liver severity in university staff. Workplace-based metabolic screening and lifestyle interventions are recommended to reduce the burden of MASLD in academic settings.

## 1 Introduction

The liver is the largest and one of the most metabolically complex organs in the human body, playing a vital role in nutrient metabolism and the detoxification of drugs and harmful substances (1). Among hepatic disorders, fatty liver disease—originally classified into Non-Alcoholic Fatty Liver Disease (NAFLD) and Alcoholic Fatty Liver Disease (AFLD)—has emerged as a major global concern. NAFLD refers to hepatic steatosis accompanied by inflammation and hepatocyte ballooning in individuals who do not consume alcohol in significant quantities (2).

In recent years, a paradigm shift in terminology has taken place, with NAFLD now redefined as Metabolic dysfunction-associated fatty liver disease (MAFLD) to better reflect the underlying metabolic disturbances rather than solely excluding alcohol use. This renaming aims to reduce the stigma associated with terms like “non-alcoholic” and “fatty,” which are often misunderstood as indicative of alcoholism or poor lifestyle choices (3). Global consensus on this nomenclature—led by major associations such as AASLD, EASL, and ALEH—was formalized in 2023, resulting in a new classification system (4).

As part of this change, Steatotic Liver Disease (SLD) has become the umbrella term for hepatic steatosis of any cause. Within this classification, MAFLD has been replaced by Metabolic dysfunction-associated steatotic liver disease (MASLD), and NASH is now referred to as Metabolic dysfunction-associated steatohepatitis (MASH) (5). Unlike NAFLD, MASLD diagnosis does not require the exclusion of other causes but rather the presence of steatosis with at least one of several defined metabolic risk factors. This change provides a more inclusive and pathophysiologically relevant framework (4).

The pathogenesis of MASLD is multifactorial, involving genetic predispositions, metabolic abnormalities, environmental influences, and gut microbiota interactions. Central obesity and insulin resistance are key drivers, though other risk factors such as hypertension, dyslipidemia, obstructive sleep apnea, hypothyroidism, and even abnormal sleep duration also contribute significantly (6). In particular, metabolic syndrome—characterized by central obesity, dyslipidemia, hypertension, and insulin resistance—has been widely recognized as a major contributor to MASLD development. In Indonesia, the prevalence of metabolic syndrome reaches 23% (7).

Moreover, work-related factors such as long years of service, prolonged sitting duration, and chronic occupational stress have also been linked to systemic inflammation and metabolic dysfunction. These conditions may promote hepatic fat accumulation through hormonal pathways, including elevated cortisol levels that contribute to insulin resistance and liver steatosis (8–10).

Globally, the prevalence of MASLD has increased markedly in the past two decades. A recent review showed a rise from 26% (1990–2006) to 38% (2016–2019), with the highest rates observed in Latin America (44.4%), followed by the Middle East and North Africa (11). In Southeast Asia, the prevalence is 33.1%, with Indonesia ranking the highest at 51.04%, ahead of Singapore (40.43%) and Malaysia (38.5%). While the global average is estimated at 25% (12), localized studies in Indonesia reveal even more alarming statistics—for instance, a study in Makassar found that 95.5% of obese patients and 67.2% of hypertriglyceridemic individuals had fatty liver disease (13).

Despite this rising trend, there is a notable absence of epidemiological data on fatty liver disease among employees in academic institutions in Eastern Indonesia. Limited research in this region hampers efforts to formulate targeted health interventions and workplace health policies (14). Most previous studies have primarily focused on the general population or clinical patients in hospital settings. However, academic staff as part of the formal, educated workforce remain underrepresented in current research—particularly in Eastern Indonesia. This is noteworthy, as chronic occupational stress and sedentary work patterns in this group may pose significant risk factors for MASLD development.

Therefore, this study aims to examine the relationship between individual characteristics—including sex, age, exercise habits, coffee and alcohol consumption, smoking habits, obesity, diabetes mellitus, dyslipidemia, and hypertension—and work-related factors such as years of service, employment status, and work unit, with the severity of fatty liver disease among university employees. The findings are expected to contribute valuable insights to the growing body of evidence on MASLD within occupational health contexts, particularly in academic work environments.

## 2 Materials and methods

### 2.1 Study design

This study employed an analytic cross-sectional design using secondary clinical data from the 2024 Medical Check-Up (MCU). This design is appropriate for assessing associations between exposures and fatty liver severity at a single point in time, without implying causal direction.

Purposive sampling was used because the sampling frame consisted exclusively of employees who had USG-confirmed fatty liver recorded in the MCU database. The study aimed to analyze determinants of fatty liver severity among confirmed cases, not to estimate prevalence in the general employee population; therefore, probability sampling was neither applicable nor methodologically required.

Because the study aimed to examine determinants of fatty liver severity among confirmed cases—not to estimate prevalence in the broader employee population—the sampling frame was intentionally limited to existing fatty liver cases. Therefore, restricting the sample to confirmed cases does not constitute selection bias, but reflects an appropriate methodological approach for analytic cross-sectional studies based on fixed clinical datasets.

### 2.2 Location and time

The study was conducted at a public university campus in Makassar, South Sulawesi, Indonesia. The research period spanned from January 2025 to July 2025, covering activities such as proposal preparation, ethical approval, data collection, analysis, and report writing.

### 2.3 Sampling and participants

A total of 78 participants were selected using purposive sampling, based on specific inclusion and exclusion criteria. The inclusion criterion was a confirmed diagnosis of fatty liver based on abdominal ultrasound (USG) findings. Inclusion was strictly based on the presence of ultrasound-confirmed hepatic steatosis documented in the 2024 MCU database. This procedure did not involve any researcher-driven selection to increase the number of MASLD cases. Rather, all eligible individuals with USG-confirmed fatty liver were included (a census of cases), ensuring that the analysis represented the entire case population within the institution. Meanwhile, the exclusion criterion was a documented history of fatty liver disease prior to their employment at the university, to ensure that all cases reflected conditions acquired during or after their occupational tenure. Other known causes of hepatic steatosis, such as liver infections or long-term use of hepatotoxic drugs, were also excluded to ensure that the observed steatosis reflected metabolic-related etiology rather than alternative pathological processes.

### 2.4 Variables

This study assessed a range of individual and work-related variables hypothesized to influence the severity of fatty liver disease. These variables were selected based on previous literature and clinical relevance. All data used in this study were obtained from the institutional MCU database, which routinely includes biometric measurements, laboratory results, and standardized abdominal ultrasonography findings for all employees. The MCU was conducted by certified clinical staff between January and March 2024. Data extraction for research purposes was carried out in February–March 2025 using a predefined extraction sheet to ensure accuracy and uniformity. Therefore, all clinical, lifestyle, and occupational variables analyzed in this study were derived directly from the MCU records, and no additional data collection procedures were performed.

#### 2.4.1 Sex

Coded as male or female based on identity listed in medical records. Previous studies have shown sex-based differences in liver fat accumulation and disease progression, potentially due to hormonal and visceral fat distribution differences.

#### 2.4.2 Age

Treated as a continuous variable and categorized into two groups: < 45 years and ≥45 years based on median distribution. Increasing age is often associated with metabolic changes that may exacerbate liver fat accumulation.

#### 2.4.3 Exercise habits

Defined as engagement in regular physical activity, based on participants' self-reported habits. Responses were categorized into “Yes” (regular exercise) and “No” (not exercising regularly). Lack of physical activity has been linked to the development of metabolic syndrome and hepatic steatosis.

#### 2.4.4 Smoking habits

Determined from participants' self-reported smoking behavior. Responses were classified as “Yes” (current or past smokers) and “No” (never smoked). Smoking has been implicated in oxidative stress and metabolic disturbances that may contribute to liver dysfunction.

#### 2.4.5 Alcohol consumption

Evaluated through self-reported drinking habits. Responses were grouped into “Yes” for participants who reported consuming alcohol and “No” for those who did not.

#### 2.4.6 Coffee consumption

Assessed based on self-reported habitual consumption. Participants were categorized into “Yes” (habitual coffee drinkers) and “No” (non-habitual drinkers). Although not directly preventing fatty liver, several studies suggest coffee may have hepatoprotective effects due to its antioxidant and anti-inflammatory properties.

#### 2.4.7 Obesity

Obesity status was determined using Body Mass Index (BMI). Participants were categorized as “Yes” if their BMI was ≥25 kg/m^2^, and “No” for all values below that threshold, based on WHO Asian criteria. Obesity, particularly central obesity, is a major driver of insulin resistance and fatty liver pathogenesis.

#### 2.4.8 Diabetes mellitus

Diagnosis of diabetes mellitus was based on blood glucose levels. Participants were categorized as “Yes” if fasting blood glucose (FBG) was ≥126 mg/dL or if 2-h postprandial glucose was ≥200 mg/dL. All others were classified as “No.” Diabetes is a well-established risk factor for MASLD and contributes to liver fibrosis progression.

#### 2.4.9 Dyslipidemia

Dyslipidemia was identified from lipid profile results. Respondents were classified as “Yes” if one or more of the following were present: total cholesterol ≥200 mg/dL, LDL ≥130 mg/dL, triglycerides ≥150 mg/dL, or HDL < 40 mg/dL (men)/ < 50 mg/dL (women). Others were categorized as “No.” Dyslipidemia is a hallmark of metabolic syndrome and plays a key role in hepatic lipid accumulation.

#### 2.4.10 Hypertension

Hypertension status was determined from blood pressure readings. Participants were labeled as “Yes” if they had a systolic pressure ≥140 mmHg and/or diastolic pressure ≥90 mmHg, or if they were using antihypertensive medication. Others were labeled “No.” Hypertension contributes to systemic inflammation and vascular changes linked to MASLD progression.

#### 2.4.11 Years of service

Calculated based on total years of employment. Categorized as ≤ 26 years and >26 years based on the median. Prolonged service may correlate with chronic occupational stress and sedentary patterns, contributing to metabolic dysfunction.

#### 2.4.12 Employment status

Categorized as civil servants or contract-based staff. This variable reflects potential differences in workload, job security, and benefits, which may influence health outcomes.

#### 2.4.13 Work unit

Participant's work units were categorized based on the nature of their job duties into academic (e.g., teaching or research-oriented roles) and non-academic (e.g., administrative or operational support roles). To maintain confidentiality, specific unit names were anonymized in this study. This classification aims to reflect potential differences in work-related stress levels, physical activity, and job demands across different functional areas within the institution.

#### 2.4.14 Fatty liver (variable dependent)

The main outcome of this study. Fatty Liver severity was determined based on abdominal ultrasonography findings and categorized into two groups: Grade 1 (mild) and Grade 2–3 (moderate–severe). This variable represents the dependent variable used to assess the influence of individual, metabolic, and occupational factors. The use of USG in this study reflects the available clinical data in the MCU records.

### 2.5 Statistical analysis

Data were analyzed using SPSS version 29. The analysis consisted of three stages:

- Univariate analysis was conducted to describe the distribution and frequency of each variable.
- Bivariate analysis used the Chi-square test to assess the relationship between each independent variable and fatty liver severity. Variables with p < 0.25 in the bivariate analysis were entered into the multivariate.
- Multivariate analysis was conducted using binary logistic regression because the outcome variable (fatty liver severity) was dichotomized into mild (Grade 1) vs. moderate–severe (Grades 2–3). This method is appropriate for identifying independent predictors while adjusting for potential confounders. Results were expressed as odds ratios (OR) with 95% confidence intervals (CI).

Multicollinearity among independent variables was assessed using Variance Inflation Factor (VIF) and tolerance statistics. All variables demonstrated acceptable VIF (< 2) and tolerance (>0.5) values, indicating no evidence of problematic multicollinearity in the regression model. Model calibration was evaluated using the Hosmer–Lemeshow goodness-of-fit test to ensure adequate model performance.

### 2.6 Ethical approval

Approved by the Health Research Ethics Committee, Faculty of Public Health, Universitas Hasanuddin (Ethical Clearance No. 943/UN4.14.1/TP.01.02/2025).

## 3 Results

A total of 78 employees with ultrasound-confirmed fatty liver disease were included. Women represented 56.4%, and most participants were aged ≥45 years (92.3%), reflecting a predominantly middle-aged to older workforce. Regarding lifestyle factors, 74.4% reported engaging in regular exercise, while all respondents denied smoking and alcohol use. Coffee consumption was reported by 6.4% of participants. For metabolic conditions, 26.9% were obese, 20.5% had diabetes mellitus, 70.5% had dyslipidemia, and 44.9% had hypertension. In terms of occupational characteristics, 51.3% had worked for more than 26 years, and 78.2% were academic staff. Most respondents (84.6%) worked in academic units (Table 1). Based on ultrasound grading, 73.1% had mild fatty liver (Grade 1), while 26.9% had moderate-to-severe disease (Grades 2–3) (Table 2).

**Table 1:** Characteristics of respondents (*n* = 78).

**Variable**	**Category**	**Frequency (*n*)**	**Percentage (%)**
Sex	Male	34	43.6
	Female	44	56.4
Age	< 45 years	6	7.6
	≥45 years	72	92.3
Exercise habit	Yes	58	74.4
	No	20	25.6
Smoking habit	Yes	0	0.0
	No	78	100.0
Alcohol consumption	Yes	0	0.0
	No	78	100.0
Coffee consumption	Yes	5	6.4
	No	73	93.6
Obesity	Yes	21	26.9
	No	57	73.1
Diabetes mellitus	Yes	16	20.5
	No	62	79.5
Dyslipidemia	Yes	55	70.5
	No	23	29.5
Hypertension	Yes	33	42.4
	No	45	57.7
Years of service	< 26 years	38	48.7
	≥26 years	40	51.3
Employment status	Lecturer	61	78.2
	Administrative staff	17	21.7
Work unit	Academic	66	84.6
	Non-academic	12	15.38

**Table 2:** Fatty liver severity distribution.

**Fatty liver grade**	**Frequency (*n*)**	**Percentage (%)**
Grade 1 (Mild)	57	73.1%
Grade 2–3 (Moderate–Severe)	21	26.9%
Total	78	100%

The bivariate analysis identified sex, obesity, dyslipidemia, and years of service as variables significantly associated with fatty liver severity (*p* < 0.05). Male sex was associated with higher odds of moderate-to-severe disease (OR = 7.154, 95% CI 1.894–27.015; *p* = 0.002). Obesity increased the likelihood nearly fivefold (OR = 4.756, 95% CI 1.001–22.551; *p* = 0.035). Dyslipidemia showed the strongest crude association (OR = 18.295, 95% CI 1.993–167.909; *p* = 0.010). Participants with ≥26 years of service showed a significant bivariate association (OR = 3.696, 95% CI 1.249–10.933; *p* = 0.015). Other individual factors—including age, diabetes, hypertension, coffee intake, and work unit—showed no significant associations in bivariate testing (Table 3).

**Table 3:** Bivariate analysis of fatty liver severity risk factors.

**No**.	**Risk factor**	**∑Mild (*n* = 57)**	**∑Moderate–Severe (*n* = 21)**	**OR**	**95% CI**	**(*P-value*)**
1	Sex					
	Male	26 (59.1%)	18 (40.9%)	7.154	1.894–27.015	0.002
	Female	31 (91.2%)	3 (8.8%)			
2	Age					
	< 45 years	5 (83.3%)	1 (16.7%)	1.923	0.211–17.497	0.556
	≥45 years	52 (72.2%)	20 (27.8%)			
3	Exercise habit					
	Yes	40 (69%)	18 (31%)	2.55	0.662–9.812	0.163
	No	17 (85%)	3 (15%)			
4	Smoking^a^					
	Yes	0	0	0	0	0
	No	57 (73.1%)	21 (26.9%)			
5	Alcohol consumption^a^					
	Yes	0	0	0	0	0
	No	57 (73.1%)	21 (26.9%)			
6	Coffee consumption					
	Yes	4 (80%)	1 (20.00%)	0.663	0.070–6.291	0.718
	No	53 (72.6%)	20 (27.4%)			
7	Obesity					
	Yes	38 (66.7%)	19 (33.3%)	4.75	1.001–22.551	0.035
	No	19 (90.5%)	2 (9.5%)			
8	Diabetes mellitus					
	Yes	10 (62.5%)	6 (37.5%)	1.88	0.585–6.040	0.285
	No	47 (75.8%)	15 (24.2%)			
9	Dyslipidemia					
	Yes	35 (63.6%)	20 (36.4%)	12.571	1.574–100.422	0.004
	No	22 (95.7%)	1 (4.3%)			
10	Hypertension					
	Yes	24 (72.7%)	9 (27.3%)	1.031	0.375–2.836	0.952
	No	33 (73.3%)	12 (26.7%)			
11	Years of service					
	< 26 years	34 (85%)	6 (15%)	3.696	1.249–10.933	0.015
	≥26 years	23 (60.5%)	15 (39.5%)			
12	Employment status					
	Academic staff (Lecturer)	41 (68.3%)	19 (31.7%)	3.707	0.773–17.773	0.085
	Administrative Staff	16 (88.9%)	2 (11.1%)			
13	Work unit					
	Academic	47 (71.2%)	19 (28.8%)	2.021	0.404–10.102	0.384
	Non-academic	10 (83.3%)	2 (16.7%)			

^a^Smoking and alcohol variables were excluded from inferential analysis due to zero responses (“No” for all participants), which prevented meaningful statistical comparison.

Binary logistic regression was performed to identify dominant factors associated with fatty liver severity. The results showed that sex, obesity, and dyslipidemia remained significantly associated in the final model.

- Male sex was found to have 10.1 times higher odds of developing moderate-to-severe fatty liver compared to females (OR = 10.167, 95% CI = 2.283–45.285, *p* = 0.002).
- Participants with obesity had 6.3 times greater odds (OR = 6.358, 95% CI = 1.010–40.028, *p* = 0.049).
- Those with dyslipidemia had the strongest association, with 18.2 times greater odds of developing more severe fatty liver disease (OR = 18.295, 95% CI = 1.993–167.909, *p* = 0.010).

**Figure 1:**
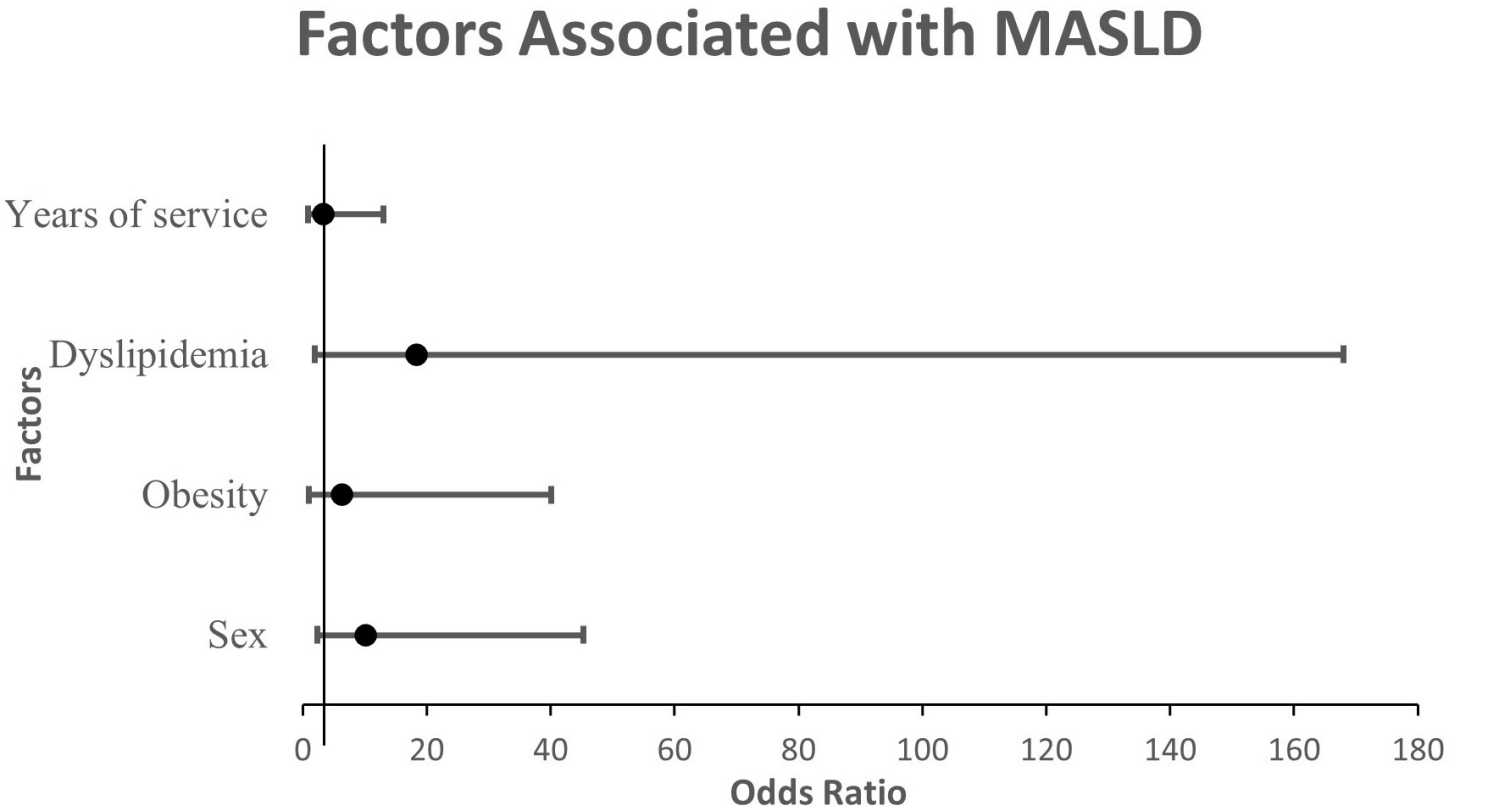
Forest plot of adjusted odds ratios (OR) and 95% confidence intervals for factors associated with MASLD severity among university employees. OR values are plotted on a linear scale.

The variable years of service were not significantly associated with Fatty Liver severity in the multivariate model (Table 4). The adjusted associations identified in the multivariate logistic regression model are illustrated in Figure 1.

**Table 4:** Multivariate analysis table.

**Variable**	***p-value* (Sig.)**	**OR [Exp(B)]**	**95% CI (Lower–Upper)**	**Conclusion**
Sex	0.002	10.167	2.283–45.285	Significant
Obesity	0.049	6.358	1.010–40.028	Significant
Dyslipidemia	0.010	18.295	1.993–167.909	Significant
Years of service	0.079	3.359	0.870–12.962	Not significant

## 4 Discussion

This study explored the relationship between individual and occupational factors and the severity of fatty liver disease among university employees. The findings revealed that male sex, obesity, and dyslipidemia were significantly associated with moderate-to-severe fatty liver, as demonstrated in both bivariate and multivariate analyses. In contrast, variables such as physical activity, years of service, and employment status did not show statistically significant associations in the final model.

Sex was a consistent predictor of fatty liver severity (*p* = 0.002; OR = 10.167; 95% CI = 2.283–45.285), indicating that male respondents were over 10 times more likely to experience moderate-to-severe fatty liver compared to females. This finding is consistent with previous studies (15, 16), which attribute the disparity to hormonal factors and fat distribution—particularly the higher accumulation of visceral fat in males, which contributes to hepatic inflammation and insulin resistance.

Obesity was also significantly associated with disease severity (*p* = 0.049; OR = 6.358; 95% CI = 1.010–40.028), reinforcing its role as a central component in the pathogenesis of MASLD. Previous studies (16–18) describe obesity as a key driver in the “multiple-hit” hypothesis of fatty liver disease, where it exacerbates other metabolic disturbances. These studies further demonstrated that BMI positively correlates with hepatic fat accumulation and that even modest weight loss (5–10%) can reduce hepatic steatosis.

Dyslipidemia emerged as the strongest independent risk factor [*p* = 0.010; OR = 18.295; 95% CI = (1.993–167.909)], with affected individuals being over 15 times more likely to develop moderate-to-severe fatty liver. Previous findings (19) noted that lipid abnormalities—especially elevated triglycerides and low HDL—strongly contribute to hepatic fibrosis in MASLD patients. These findings highlight dyslipidemia's central role not only in the onset but also in the progression of liver disease.

Years of service >26 years showed a potential association in the bivariate analysis (*p* = 0.015; OR = 3.696), but this was not statistically significant in the multivariate model (*p* = 0.079). Nevertheless, the elevated OR suggests a clinically meaningful trend. Prolonged employment may reflect cumulative exposure to sedentary behavior, work-related stress, and unhealthy lifestyle patterns. A previous study by Wang et al. (20) found that working more than 50 h per week increased MASLD risk by 57%, while Huang et al. (21) showed that night shift workers with >10 years of service had significantly higher MASLD risk. Although this study did not account for shift work, the evidence supports the impact of long-term occupational exposure on metabolic liver dysfunction.

Previous research by Siswadi et al. (22) among industrial workers showed that prolonged years of service significantly impact employee wellbeing, with high work stress mediating job satisfaction (*p* = 0.002) and linking to mental and physical workload effects on performance (*p* < 0.05). Similarly, Saleh et al. (23) found that among air-traffic controllers, longer work duration notably increased stress and psychological fatigue (*p* = 0.033 and *p* = 0.001). These findings align with our results, where >26 years of service was significantly associated with moderate-to-severe fatty liver (OR = 3.696; *p* = 0.015). However, this association was attenuated in the multivariate model. This suggests that the effect of long employment duration may be mediated through metabolic factors—particularly obesity and dyslipidemia—which are strongly correlated with prolonged sedentary work patterns and chronic occupational stress. In other words, employees with longer years of service may accumulate metabolic risks over time, but these risks are more directly captured by obesity and lipid abnormalities in the adjusted model. Thus, the lack of significance in multivariate analysis does not negate its relevance; rather, it indicates an indirect pathway where years of service contribute to MASLD severity through intermediate metabolic mechanisms.

Other individual factors such as age, physical activity, coffee consumption, diabetes mellitus, and hypertension did not show significant associations with fatty liver severity in this sample. While age is commonly considered a risk factor, our findings (*p* = 0.556) suggest that its effect may be mediated through other variables like obesity and lipid status. Similarly, despite the well-known protective effects of exercise, no significant relationship was found (*p* = 0.163). This may be due to limitations in measurement, as exercise was reported dichotomously (yes/no), without accounting for intensity, frequency, or duration—factors emphasized by as essential for hepatic improvement (24). Coffee consumption was also recorded in binary form, and only five respondents reported habitual intake, resulting in limited variability. Smoking and alcohol consumption were reported as “No” by all participants; this may reflect social desirability bias or underreporting in a professional university setting. Due to the absence of variation, these variables were not included in further inferential analysis.

Alcohol and smoking were excluded from inferential analysis due to zero responses. While this may reflect healthy habits among university employees, underreporting or social desirability bias cannot be ruled out, especially in professional or culturally conservative settings.

Coffee consumption also showed no association with fatty liver severity (*p* = 0.718). Although studies suggest hepatoprotective effects of coffee, the limited number of coffee consumers (*n* = 5) in our sample likely prevented meaningful analysis (25).

Contrary to expectations, diabetes mellitus (*p* = 0.285) and hypertension (*p* = 0.952) did not show significant relationships with disease severity. This may result from effective disease management among respondents or limitations in sample size. Two previous studies (17, 26) emphasized the roles of blood glucose and pressure control in slowing MASLD progression, indicating that clinical management may modify the impact of these comorbidities.

Employment status (academic vs. administrative) was also not significantly associated with fatty liver severity (*p* = 0.656). Despite differing job roles, similar exposure to sedentary behavior and occupational stress might contribute to shared risk profiles. Additionally, the small number of administrative staff (*n* = 17) could limit statistical power.

From a workplace perspective, most respondents were concentrated in five major units, each representing both academic and administrative functions. Overall, obesity and dyslipidemia were common across these units, while male respondents predominated in most groups. However, years of service was the only factor that showed a statistically significant difference across work units (*p* = 0.001), with employees in certain units having shorter employment durations. This finding may reflect differences in occupational exposure, work routines, and cumulative metabolic risks among university staff.

Overall, the final multivariate model confirmed sex, obesity, and dyslipidemia as independent predictors of moderate-to-severe fatty liver disease. These findings underscore the potential role of individual and workplace-related factors in MASLD progression. As depicted in Figure 1, the forest plot reinforces these results by summarizing the adjusted odds ratios and confidence intervals in a single visual framework.

This study has several limitations. First, the sample size was relatively small and drawn from a single university setting, which may limit the generalizability of the findings. These associations should be interpreted with caution, as the cross-sectional design limits the ability to infer causal relationships. The identified predictors represent correlations at a single time point rather than temporal sequences of risk factor accumulation. Second, the sampling approach relied on a fixed clinical database of employees with USG-confirmed fatty liver; while appropriate for analytic cross-sectional studies based on secondary data, selection bias and residual confounding cannot be completely excluded. Third, although ultrasonography is widely used in institutional health evaluations in Indonesia, it is not the gold-standard imaging modality for steatosis diagnosis, and the use of USG may reduce diagnostic precision compared to FibroScan. Therefore, the results should not be overgeneralized beyond this specific university population, and further studies with larger, more diverse samples are needed to validate these findings.

Fourth, several lifestyle variables were limited by the structure of the MCU database. Exercise was measured only as a Yes/No response without information on frequency, duration, or intensity, which may lead to misclassification and reduce the ability to detect associations. Coffee consumption was extremely low (*n* = 5), limiting the interpretability of its effect. Smoking and alcohol use were reported as “No” by all participants, possibly reflecting social desirability bias, which prevented meaningful analysis of these variables.

## Data Availability

The datasets presented in this article are not readily available because the dataset is confidential and owned by Universitas Hasanuddin. It cannot be shared publicly due to institutional and ethical restriction. Requests to access the datasets should be directed to Mutmainnah Husaema sora.kusanagi@gmail.com.
